# Another Anomalous Tooth

**Published:** 1842-12

**Authors:** 


*Another Anomalous Tooth.*—We described in a former number of the Journal, a tooth that had been presented to us by Dr. Jenks, dentist, of Fredericktown, Md. which had upon one of its sides, above the termination of the enamel and at the bifurcation of two of its roots, an enamelled protuberance the size of a pin's head. We at the same time stated that we had received another tooth, having on it a similarly enamelled protuberance from Mr. (but now Dr.) Savier. We have subsequently received one more, which has on it, partly between the bifurcation of two of its roots an enamelled protuberance of a much larger size than either of those on the other teeth. For this last, we are indebted to Dr. N. Dodge, dentist, of New York, who, previously to sending it to us, had it very neatly mounted upon a pedestal and covered with a glass shade. For his kindness, we beg to return him our most sincere thanks.

